# Brain cancer prognosis: independent validation of a clinical bioinformatics approach

**DOI:** 10.1186/2043-9113-2-2

**Published:** 2012-02-01

**Authors:** Raffaele Fronza, Michele Tramonti, William R Atchley, Christine Nardini

**Affiliations:** 1 Key Laboratory of Computational Biology, MPG-CAS PICB, Shanghai, China; 2 Department of Genetics, North Carolina State University, Raleigh, NC, USA

**Keywords:** glioblastoma, survival, system, emergent property, high-throughput biology

## Abstract

Translational and evidence based medicine can take advantage of biotechnology advances that offer a fast growing variety of high-throughput data for screening molecular activities of genomic, transcriptional, post-transcriptional and translational observations. The clinical information hidden in these data can be clarified with clinical bioinformatics approaches. We have recently proposed a method to analyze different layers of high-throughput (*omic*) data to preserve the emergent properties that appear in the cellular system when all molecular levels are interacting. We show here that this method applied to brain cancer data can uncover properties (i.e. molecules related to protective versus risky features in different types of brain cancers) that have been independently validated as survival markers, with potential important application in clinical practice.

## 1 Background

We have recently presented in [1] an approach to identify the so called *emergent *properties of a biological system, i.e. properties that arise from the interaction of portions of a system. In particular, this method is based on the integration of translational (microarrays for mRNA gene expression) and post-translational (RT-PCR of miRNAs) data and applied to observations related to human brain tumors published in [2]. Emergent properties are a well known concept in Systems Theory and are now becoming more common in Systems Biology [3-6]. In general, the concept of emergent property relates to the fact that a system studied in its entirety shows features that cannot be captured when the system is observed through its (simplified) subsystems (*Reductionist *approach). Applied to molecular biology, this corresponds to the observation that separate analyses of different aspects of a system (e.g., transcriptional and/or post-transcriptional mechanisms) lead to results that may not be concordant with analyses of the system as a whole. This may be due to underestimating or overlooking interactions among miRNAs and mRNAs. The identification of emergent properties can be done through the use of latent variables in multivariate statistics (in particular via the use of Factor Analysis, FA, [7]). Latent variables are so-called hidden variables which are not evident in the original observed data, because they emerge from consideration of the covariance patterns when a large number of relevant variables are analyzed simultaneously.

Taking advantage of the parallelism existing between biological systems' emergent properties and latent variables, we have used the ability of latent variables to describe emergent properties, by applying multivariate analysis simultaneously to different parts of a biological system, and notably to transcriptional and post-transcriptional data. In practice, each latent variable (i.e. each factor) obtained from analyzing jointly the mRNA and miRNA data consists of a group of heterogeneous molecules (mRNAs, miRNAs). It is then the *interaction *among molecules in the same group (i.e. factor) that defines an *emergent *property. This was done on a dataset of 330 miRNAs and ~14,500 mRNAs that for our purposes were merged (in the joint analysis) into a single table (containing all molecular data and as many clinical indications of the tumor class as there are samples, twelve) [1]. Conversely, traditional *parallel *analyses imply that the two mRNA and miRNA data tables are studied separately, and that annotation results are jointly discussed only afterwards. Therefore, the association between miRNAs and mRNAs relies solely on manual curation, while our approach offers to researchers non-trivial associations (built in the factors) that can then be manually investigated further to elucidate the exact nature of the association. Results have shown that the designed approach is more helpful than traditional approaches (that analyze distinctly the two tables of mRNA and miRNA data, or use hierarchical clustering, correlation or tools specific for differential analyses [2,8,9]) in identifying non-trivial biological properties [1]. In fact, in contrast to traditional approaches, we were able to discover the relevance of two miRNA *clusters *(miR-17-92 and miR-106-363), which appear to be important for the diagnosis of glioblastoma versus gliosarcomas. A *cluster *is a group of co-localized miRNAs, in this particular case one maps onto Chromosome X (miR-17-92) and one maps onto Chromosome 13 (miR-106-363).

Briefly, these polycistronic miRNA genes are involved in cell proliferation, apoptosis suppression, tumor angiogenesis [10] and T cell leukemia [11]. Although lying on different areas of the genome, the two clusters are closely related because each miRNA on one cluster has at least one homologue in the other cluster except for miR-17-3p and miR-363 that do not share homology with the other miRNAs. Finally, we have observed that the list of predicted targets (using the Targetscan software, [12]) is identical for all miRNAs.

## 2 Independent Validation of Findings

The present article relates and discusses the coherence of the findings in two independent publications, the one described above and reported in [1] and the independent validation published in [13], where the authors identify an innovative miRNA survival signature for Glioblastomas, based on a classical statistical approach (survival analysis), on a much larger set of data (222 glioblastomas from The Cancer Genome Atlas dataset). In more recent years miRNAs have appeared to be extremely meaningful in the evolution of tumors [14] and the results presented in [13] confirm this trend. The signature identified in [13] is composed of ten miRNAs, three of which appear to be *protective *(i.e. allowing longer survival when overexpressed), and seven are *risky *(viceversa).

In summary, the two papers we compare are related to: (i) identification of a miRNA survival signature performed with survival Cox statistics on miRNA glioblastoma data [13]; (ii) identification of emergent properties performed with factor analysis on four types of mRNA and miRNA glioma data processed in the same table [1]. The second one represents a very general question (identification of distinctive molecular characteristics of different types of tumors), and yet it is able to identify, as emergent property, the protective action of the same miRNAs highlighted in the survival analysis.

**Figure 1:**
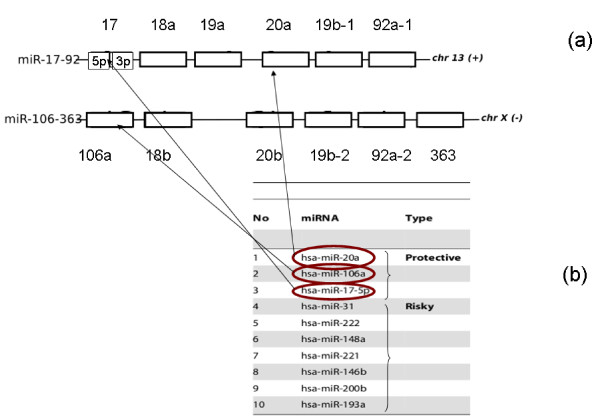
**Organization of miRNA survival related clusters miR-17-92 and miR-106-363**. Panel (a) depicts the structure of the two polycistronic miRNA genes identified in our previous work [1]. Panel (b) lists the miRNAs constituting the survival signature identified by Srinivasani et al. The protective miRNAs signature could be identified without any a priori knowledge on its role in patients' survival in [1]. Adapted from [13] and [1].

Therefore the same molecules could be isolated with both methods, and complementary advantages. The first approach [13] has a clear clinical focus with results relevant in diagnosis and prognosis, additionally, to provide sufficient statistical power to the test, this work is based on a large dataset. The second approach [1] was not guided by a specific medical nor biological question, indeed it represented an extended analysis on a much smaller dataset, originally collected to explore the connection between miRNAs and their targets in gliomas. Nevertheless, it was able to extract clinically relevant information. In fact, the protective markers identified in [13] (namely hsa-miR-20a; hsa-miR-106a; hsa-miR-17-5p) all lie on the clusters miR-17-92 and miR-106-363 identified by our analysis in [1]. Figure 1 depicts the relationship between the two sets of results.

## 3 Discussion

In our previous paper [1] we reported that the involvement of cluster miR-17-92 is related to solid tumor angiogenesis, and since the associated factor is related to the discrimination of gliosarcomas from other brain tumors, we concluded that cluster miR-17-92 (and its homologous-rich companion, cluster miR-106-363) could be involved in the development of the sarcomatous element. In fact, despite the poor prognosis, gliosarcomas generally allow a longer survival time than glioblastomas [15] due to the protective sarcomatous component of the tumor [16,17]. Overall, since the sarcomatous element is regulated by miR-17-92 and miR-106-363, these clusters can be associated to better survival: this is now independently confirmed by [13].

Additionally, we can speculate further on the role of mir-193a. This is identified in [13] as a risk factor, meaning that its overexpression leads to shorter survival. In our analysis [1] mir-193a appears to be negatively associated to the factor that characterizes less aggressive tumors. This mathematical feature (the negative sign of mir-193a in the factors *Loadings *matrix, [7]) translates into biological terms as less aggressive tumors being associated to diminshed activity of mir-193a, from which, we can indirectly infer that its over activity is, if anything, a risk factor.

Globally, the coherence of the results obtained in [1] and in [13] highlighted in the present article, namely the relevance of the polycistronic miR-17-92 and miR-106-363 miRNA genes, is promising in two main respects.

First, the results from both papers [1,13] confirm the importance of the polycistronic clusters in clinical practice, for their ability to predict better prognosis, and consequently to better tailor patients' therapy. Second, it offers a useful analysis tool in the clinical bioinformatics research area. In fact, the dropping costs of high-throughput technologies allow many laboratories to have access to *omic *transcriptional and post-transcriptional screens, either directly generated or downloaded from public repositories. One example for all is the work being done by multiple labs on the NCI-60 cancer cell lines (http://discover.nci.nih.gov/cellminer/home.do) for which different laboratories have produced different *omic *data layers (mRNA and miRNA in [18] or mRNA and proteins in [19]). In this scenario it becomes natural to consider the possibility to merge (or generate missing layers and then merge) different data layers (mRNAs, miRNAs, proteins, etc.) to obtain more information than the analysis of one single layer can give. The type of information obtained can be used to dig into the molecular features of different subtypes of cancers (see classical approaches like [20-22]), or to associate molecular with phenotypic and clinical features [1,23]. Our joint approach is tailored for the above depicted scenario, where different types of data are merged. In particular, our approach is useful to extract information beyond the results that can be obtained by expression profile correlation and by clustering or SAM analysis applied to each omic layer independently.

## Authors' contributions

RF and CN analyzed the results, MT and WRA contributed to the validation. All authors read and approved the final manuscript. This work is funded by the National Science Foundation of China (NSFC), grant n. 31070748.

## Competing interests

The authors declare that they have no competing interests.
